# A Case of Primary Ewing Sarcoma of the Kidney: Robotic-Assisted Nephron-Sparing Surgery, a Feasible Alternative in Treatment of Localized Disease

**DOI:** 10.3390/curroncol31100443

**Published:** 2024-10-02

**Authors:** Amr Ahmed, Aleksa Zubelic, Milan Radovanovic, Gjoko Stojanoski, Metin Aksünger

**Affiliations:** 1 Department of Urology, Varisano Kliniken Frankfurt-Main-Taunus, 65812 Bad Soden, Germany; amr.ahmed@varisano.de (A.A.); gjoko.stojanoski@varisano.de (G.S.); metin.aksuenger@varisano.de (M.A.); 2 Clinic of Urology, University Clinical Centre of Serbia, 11000 Belgrade, Serbia; milan.radovanovic@med.bg.ac.rs

**Keywords:** Ewing sarcoma, nephron-sparing surgery, robotic-assisted

## Abstract

Extra-skeletal Ewing sarcoma (EWS) occurs in about 12% of EWS patients; at the same time, primary involvement of the kidneys remains extremely rare. Since it was first described in 1975, only a small case series have been reported worldwide. About 95% of surgically treated patients with EWS of the kidney described in the literature underwent nephrectomy, and the remaining patients only had a tumor biopsy. Nephron-sparing surgery (NSS) has not been sufficiently investigated as an alternative in the local surgical treatment of localized disease, mostly as a result of technically unfeasible provisions of negative surgical margins. In this report, we present a unique case of primary EWS of the kidney with an asymptomatic course without radiographic signs that suggest a highly aggressive disease, successfully locally treated with robotic-assisted NSS. This report showcases that robotic-assisted NSS could be a feasible alternative in treatment of localized disease yielding equally good oncological results while, at the same time, creating better prerequisites for necessary adjuvant chemotherapy.

## 1. Introduction

Primary Ewing sarcoma (EWS) of the kidney is a rare tumor in adults. Since it was first described in 1975, only a small case series of primary renal Ewing sarcoma have been reported worldwide, with a summary of 116 cases diagnosed at a median age of 27–28 years old in a published meta-analysis [1]. Primary kidney EWS presents as a highly malignant tumor with rapid growth [2]. The clinical presentation is not specific and EWS of the kidney can appear initially as asymptomatic. Late symptoms include flank pain and hematuria, similar to other renal masses. About 66% of patients present with distant metastasis at the time of seeking medical aid, and the most common site of metastasis are lungs followed by liver and bone. Patients with metastatic disease have poor survival with a four-fold increase in relative risk of death [2,3]. No radiographic features indicative of the pathologic diagnosis have been identified. Thus, diagnosis is only possible using histopathology, immunohistochemical characteristics (IHC), and cytogenetic study. Histopathological findings typically show small round monomorphic cells with Homer Wright rosettes, and IHC is strongly positive for CD99 and FLI-1 [4]. Patients are usually treated using the similar strategy to Ewing sarcoma in the bone, namely, a combination of surgery and chemotherapy with radiotherapy being reserved for locally advanced disease. About 95% of surgically treated patients with EWS of the kidney described in the literature underwent nephrectomy, and the remaining patients only had a biopsy [1]. Nephron-sparing surgery (NSS) has not been sufficiently investigated as an alternative in the local surgical treatment of localized disease, mostly as a result of technically unfeasible provision of negative surgical margins. To the best of our knowledge, to this day there are only three cases successfully treated with NSS [5,6]. This case report illustrates that EWS can be, when detected early, successfully treated with robotic-assisted NSS and adjuvant chemotherapy, thus providing additional insight into robotic-assisted NSS as a meaningful alternative to radical nephrectomy in the local therapy of localized EWS.

## 2. Case Report

**Figure 1:**
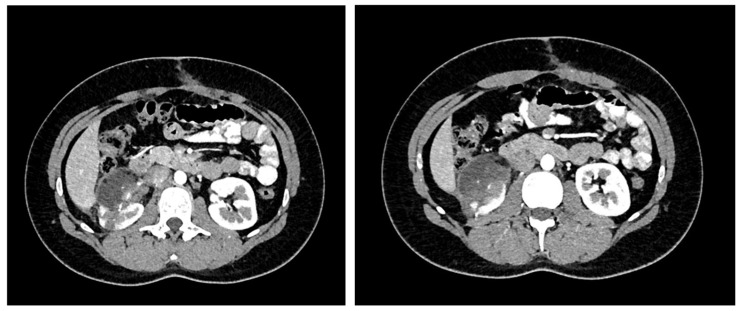
Preoperative computerized tomography (CT): Interpolarly localized isodense 6.8 cm × 6.2 cm tumor propagating to the renal sinus.

A 26-year-old man first presented himself to the outpatient unit for a routine pre-employment physical. The presence of hematuria, dysuria, flank pain, fevers, or weight loss were denied, as well as the presence of underlying disease or any current medication used. Medical history was characterized by successfully treated Hodgkin’s lymphoma (ABVD regimen and radiotherapy) 13 years ago. The physical exam, urine tests, and complete blood count were unremarkable. Abdominal sonography was routinely performed and showed an isoechoic, well-circumscribed, centrally located 6 cm × 6 cm tumor mass in the right kidney. A computerized tomography (CT) scan was indicated and confirmed an interpolarly localized isodense 6.8 cm × 6.2 cm tumor that propagated to the renal sinus and pushed the calyces for the middle pole. Gerota’s fascia and the renal vein were without signs of infiltration. Tumor showed weak to moderate opacification (with 40 to 60 HU) in the post-contrast tomograms. Hypovascular renal cell carcinoma was initially suspected (Figure 1). Consequently, a core needle biopsy was not performed.

**Figure 2:**
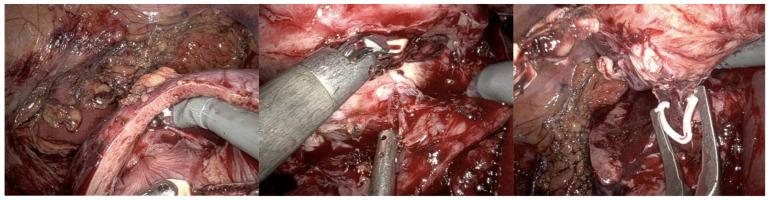
Intraoperative findings: tumor was clearly demarcated and resected from the surrounding renal tissue.

**Figure 3:**
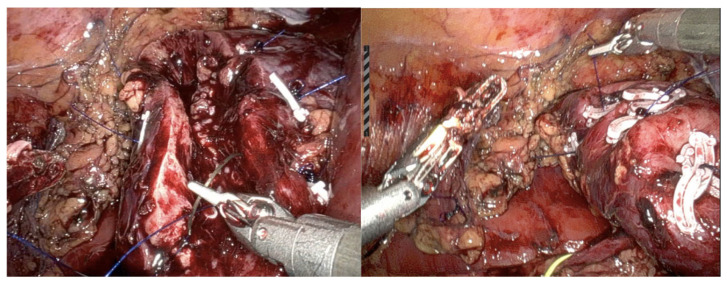
Tumor bed after removing the tumor and closing the pyelocaliceal system, as well as suturing the remaining kidney tissue.

The patient was subsequently admitted to the robotic surgery center for further operative treatment. The patient underwent an elective right robotic-assisted partial nephrectomy. Surgery was performed using a transperitoneal approach. After free preparation and in order to achieve optimal access and mobilization of the right kidney on the vascular pedicle, clamping of renal artery was performed. The tumor was encapsulated and could be clearly demarcated and resected from the surrounding renal tissue (Figure 2). The remaining renal tissue and the pyelocaliceal system were then carefully closed (Figure 3). An enlarged hilar lymph node observed intraoperatively was also removed. Warm ischemia time was 21 min, with a total robotic console operating time of 50 min. Estimated blood loss was 160 mL. Postoperative course remained uneventful, with hospitalization time of 5 days.

**Figure 4:**
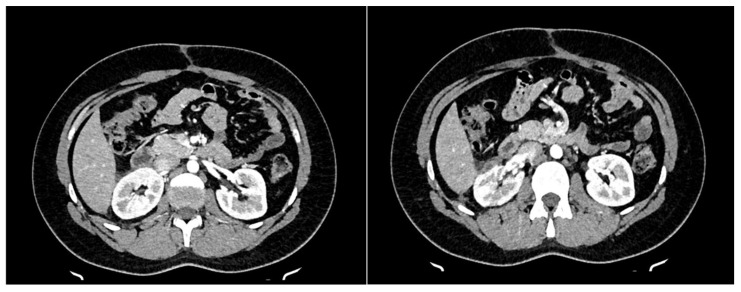
Postoperative CT-Scan showing no signs of local recurrence, in addition, small perirenal seroma can be seen.

Postoperative tumor pathology demonstrated negative surgical margins without extracapsular tumor spread into the adjacent perirenal fatty tissue. Expression of CD99 and cytoplasmic co-expression of CD117 was observed. Additional immunohistochemical tests showed negativity of the tumor cells for the S100, Melan A, and HMB45, as well as for Oct3/4 and PLAP. A weak-to-moderate variable expression of Caveolin1 with negativity of ERG, ALK, SS18, STAT6, Pan-TRK, NUT, and MUC4 and regular expression of SMARCB1 was noted. The molecular analysis, carried out in parallel with the Tru-Sight RNA Fusion Panel from Illumina, revealed a relatively rare TAF15:FLI1 fusion. Diagnosis of primary Ewing sarcoma of kidney was established. Histological examination of the removed hilar lymph node showed no signs of tumor dissemination. The patient was discharged and the multidisciplinary uro-oncology team decided to start a total of 6 courses of vincristine, ifosfamide, doxorubicin, and etoposide (VIDE–Protocol) with additional admission of Granulocyte-colony stimulating factor (G-CSF) in accordance with standardized treatment protocols for Ewing sarcoma. Chemotherapy was administered without major complications. Adjuvant radiotherapy was not indicated as negative resection margin were surgically achieved. CT controls were carried out within 3 and 6 months, and PET/CT control was carried out 9 months after surgery. Local recurrence and distal metastatic lesions could not be detected (Figure 4).

After the 9-month follow-up, the patient was well and showed no evidence of disease. Written informed consent was obtained from the patient for the publication of this case report and all the accompanying images.

## 3. Discussion

EWS is a rare pediatric malignancy of bone and soft tissue, and although extra-skeletal EWS occurs in about 12% of patients, primary involvement of the kidneys is extremely rare [7]. The cellular origin of EWS remains controversial, although it is speculated that it arises from neuroectodermal cells or primitive mesenchymal stem cells [8]. Due to its rarity, EWS is often misdiagnosed as other more common kidney cancers. Considering its aggressive progression and lower survival rate, EWS should always be included in differential diagnosis of renal masses in younger patients. Almeida et al. analyzed a published case series of kidney EWS and showed that the ratio of males to females is 3:2, while the median age at diagnosis is 30.7 years [9]; similar data regarding the predominance of males in the patient population and a median age of 26.5 at diagnosis was shown by another recently published case series [10]. A study involving 48 patients with EWS showed that 73% presented initially with acute flank pain mimicking renal stone colic with or without hydronephrosis. The published meta-analysis defined pain (54%), hematuria (29%), and bulky renal mass (28%) as the most common symptoms when presenting to the hospital [1]. Due to its aggressive nature, patients with EWS usually present with already advanced disease and metastases. Completely asymptomatic, early-diagnosed cases like ours remain relatively rare in the published case series [10]. Other published cases also show disturbances in the blood count at the onset of the first symptoms, and primarily reduced hemoglobin and red blood cell count, which was also not the case in our patient [11].

Kidney EWS can be particularly difficult to diagnose by imaging alone, as no specific signs of EWS in transabdominal US, CT, or magnetic resonance imaging have been described. The imaging characteristics of most renal EWS are indistinguishable from those of RCC [9]. Hu et al. showed that the typical manifestation of EWS is a large heterogeneous soft tissue density mass, with a specific “septum-like” enhancement in a contrast-enhanced scan. The maximum diameter of the tumor is usually greater than 10 cm. This is in complete contrast to our case, showing that even in the case of a homogeneous clearly circumscribed tumor mass with significantly smaller diameter, EWS could still be a potential differential diagnosis [12]. The role of preoperative biopsy of kidney EWS remains controversial. Some authors think that preoperative biopsy should be considered in order to early identify these tumors and to allow for delivery of neoadjuvant chemotherapy [13]. Others, on the other hand, stated that this approach would only delay the necessary surgical resection if the tumor is localized. In locally advanced or metastatic disease, a biopsy undoubtedly plays an important role [14]. The absence of suspicious features on imaging as well as the surgical resectability of the tumor mass were the reason for the absence of a preoperative biopsy in our case. Most of the cases described in the literature that were successfully treated operatively were cases where radical nephrectomy was performed. On the other hand, NSS was proven feasible decades ago for patients with imperative indications to preserve maximum kidney function, among others in the group of patients requiring adjuvant chemotherapy. As is well known, chemotherapy regimens are often accompanied by negative side effects on renal function that may have serious consequences on future kidney function, but the implications concerning cancer treatment options, eligibility for clinical trials, and overall survival are also very important [15]. Safe surgical resectability of the tumor should be a prerequisite for undertaking NSS, whereby we believe that the transperitoneal robotic-assisted approach allows for better assurances in providing negative surgical margins, as was the case in our patient. In that case, adjuvant radiotherapy is not necessary [14]. Chemotherapy modalities used in the treatment of renal EWS are mostly taken from protocols for the treatment of osseus Ewing sarcoma. They are mostly based on agents such as vincristine, cyclophosphamide, ifosfamide, etoposide, actinomycin, and doxorubicin. Very encouraging oncological results involving cancer-specific survival rate of 75% at 5 years were shown in a recently published case series using the VAC/IE regimen [10]. In our case, six courses of vincristine, ifosfamide, doxorubicin and etoposide (VIDE–Protocol) were the treatment of choice. EWS has an unusual genetic characteristic that is reflected in balanced chromosomal translocation that joins the N-terminus of EWS (on chromosome 22) with a gene from the C-terminus of the E-twenty-six (ETS) family of transcription factors (FLI1, ERG, ETV1, ETV5, and FEV). The involvement of the EWSR1 gene has been reported in approximately 90% of cases of EWS, with the EWSR1::FLI1 fusion being the most frequent [16]. TAF15::FL1 fusion, as seen in our case, was not previously described in published case series. This rare variation could potentially be associated with a milder clinical presentation of the disease, as described in our case.

## 4. Conclusions

Upfront surgical resection with adjuvant chemotherapy remains the standard procedure in the treatment of localized EWS of the kidney. Our unique case shows that kidney EWS can have an asymptomatic course without radiographic signs that impress a highly aggressive disease. In such cases, robotic-assisted NSS can yield equally good oncological results while, at the same time, creating better prerequisites for adjuvant chemotherapy.

The main limitation of this case report remains the relatively short postoperative follow-up time of 9 months, which confirms the feasibility but still does not prove the efficiency of our surgical approach. Larger case series with longer follow-up times are necessary to standardize the surgical approach to EWS, as well as to examine the role of the rare TAF15::FL1 gene fusion described in our case in the prognosis and future therapy planning.

## Data Availability

The data presented in this study are available in this article.
